# Imaging Cerebral Activity in Amyotrophic Lateral Sclerosis

**DOI:** 10.3389/fneur.2018.01148

**Published:** 2019-01-08

**Authors:** Malcolm Proudfoot, Peter Bede, Martin R. Turner

**Affiliations:** ^1^Nuffield Department of Clinical Neurosciences, University of Oxford, Oxford, United Kingdom; ^2^Computational Neuroimaging Group, Academic Unit of Neurology, Trinity College Dublin, Dublin, Ireland; ^3^Wellcome Centre for Integrative Neuroimaging, University of Oxford, Oxford, United Kingdom

**Keywords:** amyotrophic lateral sclerosis, motor neurone disease, biomarker, neuroimaging, neurophysiology, cortex

## Abstract

Advances in neuroimaging, complementing histopathological insights, have established a multi-system involvement of cerebral networks beyond the traditional neuromuscular pathological view of amyotrophic lateral sclerosis (ALS). The development of effective disease-modifying therapy remains a priority and this will be facilitated by improved biomarkers of motor system integrity against which to assess the efficacy of candidate drugs. Functional MRI (FMRI) is an established measure of both cerebral activity and connectivity, but there is an increasing recognition of neuronal oscillations in facilitating long-distance communication across the cortical surface. Such dynamic synchronization vastly expands the connectivity foundations defined by traditional neuronal architecture. This review considers the unique pathogenic insights afforded by the capture of cerebral disease activity in ALS using FMRI and encephalography.

## Introduction

Neurodegenerative disorders are increasingly understood as a disintegration of complex cerebral functional networks (1). Amyotrophic lateral sclerosis (ALS) is characterized by loss of upper and lower motor neurones of the corticospinal tract, brainstem, and spinal anterior horns, resulting in progressive weakness of downstream muscles. In addition to protean upstream causes (2, 3), there is firmly established clinical, pathological, and genetic overlap of ALS with frontotemporal dementia (FTD). The diagnosis remains a clinical one, with a lack of biomarkers being a significant barrier to the development of highly-effective disease-modifying therapy. Advanced neuroimaging, in combination with histopathological insights, has brought the brain to the forefront of biomarker development (4).

The earliest studies of cerebral blood flow in ALS employed positron emission tomography, and demonstrated a widened region of cortical activation in response to a simple motor task (5). Among the hypotheses for this “boundary shift effect” was loss of local inhibitory GABA-ergic interneuronal circuits [reviewed in (6)]. A consistent pathological feature of ALS has been the observation of increased cortical excitability, possibly reflecting reduced local inhibitory influences, measured using short-interval paired transcranial magnetic stimulation (TMS) (7, 8). Through the characterization of monogenetic associations, ALS research has expanded to include the study of what is now thought to be a long presymptomatic phase (9), in which cortical functional abnormalities may be the among the earliest detectable manifestations (10).

Blood oxygenation level-dependent (BOLD)-based functional (FMRI), with the major advantages of avoiding ionizing radiation and the greater availability of MRI technology, confirmed a profound alteration in cortical activity inherent to the pathogenesis of ALS. Tools to study cortical neurophysiology in real-time have also undergone significant development in both sensitivity and analysis. This review will consider the unique insights that FMRI and encephalography bring to the understanding of the pathogenesis of ALS at the systems level, which is increasingly of greatest relevance to the long-term goal of neuroprotective therapy.

## Functional MRI

FMRI has been extensively used to characterize network dysfunction in ALS in cross-sectional, longitudinal, and presymptomatic study designs. The practical advantages of FMRI in ALS include the widespread availability of MRI platforms, the large number of freely available analysis suites and the ability to provide crucial biological insights in relatively simple, cost-effective, and non-invasive study designs (11). Despite some inconsistencies in the literature (12), two main themes have consistently emerged from the application of FMRI to ALS: (i) the characterization of compensatory changes, such as the recruitment of additional cortical (13–16), subcortical (14, 17, 18), and cerebellar (17, 19) regions to execute motor and cognitive tasks, and ii) the concept of inhibitory dysfunction as a key facet of ALS-associated pathophysiology (6, 20).

### Methodological Considerations

Functional MRI has been extensively used to characterize network dysfunction in ALS in cross-sectional, longitudinal, and presymptomatic study designs (21) (Table 1), but method-associated limitations are rarely articulated. The protracted hemodynamic response to neural activity hampers the temporal resolution of fMRI. Blood oxygen-level dependent (BOLD) signal typically peaks only 5–6s after focal activation therefore careful study designs are indispensable for meaningful temporal inferences (22, 23). Spatial distortions and signal dropout due to susceptibility gradients near air-tissue interfaces lead to decreased BOLD signal in lateral parietal, orbitofrontal and dorsolateral prefrontal regions necessitating meticulous sequence optimization before data acquisition (24–26). Scanner noise may also impact on the interpretation of BOLD signal, particularly in the default-mode network (27), and careful experimental designs are required to minimize the influence of background noise (28). While fMRI findings are often presented by overlaying activation maps upon high-resolution structural images, the inherent spatial resolution of fMRI is limited by the signal-to-noise ratio profile of consecutive, rapid whole-brain imaging. In the majority ALS studies, the voxel size of fMRI protocols is two to four times larger than what is used for structural acquisitions (21).

**Table 1:** Selected motor task-based and resting-state fMRI studies in ALS.

**Authors**	**Year**	**Study design**	**ALS (n)**	**Controls (n)**	**Main study findings/interpretation**
Li et al. (29)	2009	Motor task: swallowing, Cross-sectional study	10	10 HC	Reduced somatosensory cortex activation in patients with dysphagia
Mohammadi et al. (30)	2009	Motor: tongue movement, Cross-sectional study	22	22 HC 5 DC (SBMA)	ALS patients with bulbar symptoms showed decreased cortical and thalamic activation
Palmieri et al. (53)	2010	Emotional attribution and recognition task, Cross-sectional study	9	10 HC	Altered emotional processing similar to patents observed in FTD.
Lule et al. (55)	2010	visual, auditory and somatosensory stimulation, Cross-sectional study	14	18 HC	Decreased response in secondary visual areas in ALS, delayed response in secondary auditory areas, reduced response to somatosensory stimulation
Goldstein et al. (52)	2011	Cognitive task, Cross-sectional study	14	8 HC	Increased left temporal and decreased precentral and left medial frontal activation: altered inhibitory processing in ALS
Kollewe et al. (13)	2011	Motor task: hand and tongue movement, Cross-sectional study	20	20 HC	Decreased cortical activation during tongue movements in patients with bulbar symptoms. Increased activation during hand movements. Different functional reorganization in limb and bulbar impairment.
Mohammadi et al. (14)	2011	Motor task, Cross-sectional study	22	22 HC	Patients stratified into three groups based on disability, Increased activation in early-stage, decreased activation in later stage disease.
Poujois et al. (15)	2013	Hand motor task Motor imagery, Cross-sectional study	19	13 HC	Motor execution and imagery yields to similar activation patterns. Increased contra- and ipsilateral somatosensory cortex activation.
Passamonti (59)	2013	Emotional processing task, Cross-sectional study	11	12 HC	Increased activation in prefrontal areas and altered amygdala-prefrontal cortex connectivity in ALS, suggestive of limbic system dysfunction
Witiuk et al. (57)	2014	Antisaccade task with eye tracking, Cross-sectional study	12	12 HC	ALS patients make more antisaccade direction errors and exhibit reduced DLPFC activation compared to controls i.e. deficits in automatic response inhibition are associated with impaired DLPFC activation
Stoppel et al. (43)	2014	Go/No-Go paradigm, Longitudinal design	14	14 HC	Increased motor activation compared to controls with subsequent decline on follow-up scanning suggestive of failing adaptive compensation
Mohammadi et al. (61)	2015	Movement inhibition task (go/no-go), Cross-sectional study	17	17 HC	Increased motor inhibition and execution related activation in patients with ALS compared to controls.
Jelsone-Swain et al. (36)	2015	Action Observation and Execution task, Cross-sectional study	19	18 HC	Increased activation during action-execution and observation in ALS patients in opercular, premotor and primary motor regions. Mirror neuron system mediated compensation.
Li et al. (35)	2015	Action observation paradigm, Cross-sectional study	30	30 HC	Action observation activates similar networks to action execution. Increased activation observed in the DLPFC and supplementary motor regions of ALS patients.
Aho-Ozhan (60)	2016	Cognitive task Cross-sectional study	15	14 HC	Impaired processing of negative emotions such as fear and disgust in ALS
Vellage et al. (54)	2016	Cognitive task: working memory Cross-sectional study	14	14 HC	Reduced hemodynamic responses in the left occipital cortex and right prefrontal cortex in ALS patients compared to healthy controls
Keller et al. (58)	2018	Cognitive task: ToM and executive task, Cross-sectional study	65	33	Increased activation in all ALS patients compared to HC. High performing patients exhibit more activation than those with neuropsychological deficits suggestive of compensation.
Mohammadi et al. (66)	2009	Resting-state fMRI, Cross-sectional study	20	20 HC	ICA analyses: decreased DMN activation in the anterior and posterior cingulate and parietal regions
Jelsone-Swain et al. (84)	2010	Resting-state fMRI, Cross-sectional study	20	20 HC	ROI analyses: decreased functional connectivity between the right and left motor cortices
Douaud et al. (20)	2011	Resting-state fMRI, Cross-sectional study	25	15 HC	Increased SMN, premotor, prefrontal and thalamic functional connectivity, interpreted as compensation and inhibitory dysfunction
Agosta et al. (42)	2011	Resting-state fMRI, Cross-sectional study	26	15 HC	Increased SMN, cingulate, cerebellar connectivity interpreted as compensation.
Fekete et al. (76)	2013	Resting state fMRI, Cross-sectional study	40	30 HC	Widespread motor, cerebellar and basal ganglia functional connectivity alterations in the ALS cohort. Accurate subject classification using multiple kernel learning.
Zhou et al. (82)	2013	Resting-state fMRI, Cross-sectional study	12	12 HC	Positive correlation between disability and functional connectivity
Agosta et al. (65)	2013	Resting-state fMRI, Cross-sectional study	20	15 HC	ICA analyses: Increased parietal connectivity is associated with cognitive deficits which may represent compensation
Welsh et al. (67)	2013	Resting state fMRI, Cross-sectional study	32	31 HC	Machine learning (support-vector machine) based on fMRI metrics achieves over 71% accuracy for disease state classification
Zhou et al. (73)	2014	Resting state fMRI, Cross-sectional study	12	12 HC	Decreased regional brain synchrony in the superior medial SMN detected by regional coherence measures
Meoded et al. (38)	2015	Resting state fMRI, Cross-sectional study		14 HC 16 PLS	Increased functional connectivity between the cerebellum and cortical motor areas and between the cerebellum and frontal and temporal cortex in primary lateral sclerosis
Schmidt et al. (77)	2014	Resting state fMRI, Cross-sectional study	64	27 HC	A strong positive correlation exist between changes in SC and FC averaged per brain region; suggesting that structural and functional network degeneration in ALS is coupled
Chenji et al. (75)	2016	Resting state fMRI, Cross-sectional study	21	40 HC	Increased DMN and reduced SMN connectivity associated with greater disability interpreted as inhibitory dysfunction
Zhou et al. (69)	2016	Resting state fMRI, Cross-sectional study	44	44 HC	Increased cerebellar, occipital and prefrontal degree centrality (DC) and decreased DC in the primary motor cortex and sensory motor regions of ALS patients
Menke et al. (79)	2016	Resting state fMRI, Presymptomatic study design	12	12 psALS 12 HC	Increased FC between the cerebellum and precuneus- cingulate-frontal lobe network in asymptomatic mutation carriers compared to controls
Trojsi et al. (72)	2017	Resting state fMRI, Cross-sectional study	21	15	Decreased FC in DMN, salience and fronto-parietal network. More significant SLN connectivity changes observed in bulbar onset patients compared to those with spinal onset.
Zhang et al. (74)	2017	Resting state fMRI, Cross-sectional study	38	35 HC	Impaired interhemispheric functional connectivity eidenceed by voxel mirrored homotopic connectivity (VMHC) reductions, correlations with CC diffusivity metrics
Zhang et al. (166)	2017	Resting state fMRI, Cross-sectional study	25	25 HC	Reduced occipital surface-based local gyrification index (LGI) is associated with decreased functional connectivity in the bilateral precuneus.
Lee et al. (80)	2017	Resting state fMRI, Presymptomatic study design		13 psALS 46 HC	Connectivity deficits detected in salience, sensorimotor, default mode and thalamic networks in presymptomatic *C9orf72* carriers
Li et al. (68)	2018	Resting state fMRI, Cross-sectional study	38	35 HC	Graph theory method (functional connectivity density FCD) Decreased FCD in the primary motor cortex, increased long-range FCD in the premotor cortex in ALS patients.
Bueno et al. (167)	2018	Resting state fMRI, Cross-sectional study	20	15 HC	Focus on Papez circuit integrity. Decreased functional connectivity in ALS patients between hippocampal, parahippocampal and cingulate regions.
Menke et al. (39)	2018	Resting state fMRI, Longitudinal study	13	3 PLS	Multi-timepoint structural-functional study, ICA and DRA, decreased FC between SMN and frontal pole, increased FC between primary motor cortex and fronto-parietal network

*HC healthy control, DC Disease Control, DLPFC DorsoLateral PreFrontal Cortex, FC functional connectivity, SC Structural Connectivity, SBMA Kennedy's disease, FTD FrontoTemporal Dementia, PLS Primary Lateral Sclerosis, DMN Default Mode Network, SMN SensoriMotor Network, ToM Theory of Mind*.

### Motor Paradigms

Pioneering FMRI studies in ALS relied initially on hand movement paradigms (15, 16), which were gradually complemented by innovative bulbar studies (13, 29, 30). In motor-task FMRI studies, different strategies have been utilized to control for limb weakness, motor effort and lower motor neuron involvement for the interpretation of cerebral activation. Motor imagery (31) has attracted considerable attention, not only for emerging brain-machine interface applications (32) but also as an FMRI paradigm for a condition like ALS in which patients typically develop severe motor disability (33). The execution and imagination of specific movements manifest in similar activation patterns in ALS and controls (15) suggesting that this approach may be particularly pertinent to patient cohorts with mixed disability profiles. Some ALS studies however report divergent activation maps in motor imagery and execution (34). Similarly to motor imagery, action observation is also thought to result in comparable cortical activity to action execution which has been used to study the mirror-neuron system in ALS (35, 36). Another approach to control for motor disability and establish ALS-specific activation patterns is the inclusion of disease-controls, i.e., non-ALS patients with motor disability (16, 37). Very few FMRI studies to date have specifically evaluated functional changes in other rarer motor neurone disorders such as the upper motor neurone-only primary lateral sclerosis (PLS) (38, 39) and lower motor neurone-dominated Kennedy's disease (30) using motor paradigms. Patient stratification into separate study groups based on motor disability is another strategy to interpret functional alterations in the context of disability (14). In light of the fundamentally divergent study designs, the inclusion of patients in different stages of their disease and small sample sizes, the inconsistent findings of motor activation studies are not surprising. Whilst, hypo- (29, 30, 40) and hyper-activation (13, 14, 16, 35, 36) of the somatosensory cortex have both been reported in response to motor tasks, the recruitment of premotor areas is a relatively consistent finding. An integrative explanation of the seemingly divergent findings is that the initial hyper-activation represents an early-stage adaptive process to execute movement (14), which gradually gives place to hypo-activation as progressive structural changes ensue (41, 42). Robust multi-timepoint longitudinal studies are required to clarify the timeline of functional changes in ALS as very few task-based longitudinal FMRI studies have been published to date (33, 43). One longitudinal study identified reduced motor activation on 3-month follow-up which was interpreted as compensatory failure due to progressive neural loss (43), while another study reported increased precentral gyrus activity 6-month after initial scanning as evidence of ongoing adaptation (33). In addition to compensatory processes in motor, premotor and supplementary motor areas (44), evidence also exist that the basal ganglia (17, 18, 45, 46), the ipsilateral motor cortex (14, 47), and the cerebellum (17, 19, 47, 48) also contribute to adaptive network reorganization.

### Extra-Motor Studies

With the increasing recognition of cognitive impairment in ALS (49, 50), a series of elegant language (51), executive (52), theory-of-mind (36), and memory (43, 53, 54) task-based activation studies have also been published. In addition to the cognitive activation paradigms, visual, auditory and somatosensory stimulation studies have further characterized the spectrum of extra-motor involvement in ALS (55, 56). Other innovative non-motor activation studies in ALS include an anti-saccade study with concurrent eye tracking to investigate dorsolateral prefrontal cortex (DLPFC) function (57). Similar to the divergent findings of motor-task studies, increased activation (36, 52, 58, 59) and impaired activation (51, 60) have both been noted on cognitive tasks, which is likely to represent stages of successful and failing adaptation. More often however a pattern of coexisting hypo- and hyper- activation is reported (37, 54, 61).

### Resting-State Studies

The analysis of task-free BOLD signal in the so-called resting-state (rsFMRI) benefits from fast acquisition times with a data-driven, more consistent experimental design, making them an attractive add-on to high-resolution structural protocols. With the establishment of the internationally collaborative Neuroimaging Society in ALS (NiSALS) (62) and successful multi-site initiatives (63), there is interest in FMRI sequence harmonization and potential for multicentre data pooling (12, 64). rsFMRI studies differ considerably in their analysis approaches and their methods span from independent component analysis (65–67), to graph theory (68, 69) and amplitude of low frequency fluctuation (ALFF) (70, 71). rsFMRI studies in ALS identified decreased frontotemporal (72), sensorimotor (70, 73–75), and cortical-subcortical (76) network integrity and increased default mode network (75), and cerebellar (38, 69) connectivity. Large combined structural-FMRI studies suggest that patterns of structural degeneration overlap with functionally impaired regions and that a strong positive correlation exists between functional and structural connectivity alterations (77). Longitudinal rsFMRI studies indicate declining functional connectivity in sensorimotor, thalamic, and visual networks and increasing connectivity in fronto-parietal and temporal circuits (39). Multimodal, structural-functional, multi-timepoint longitudinal studies (39) are best suited to characterize the natural history of progressive neurodegenerative changes (78). Data from presymptomatic carriers of ALS-causing gene mutations revealed increased cerebello-cerebral functional connectivity (79) and decreased salience, sensorimotor, default-mode, and thalamic networks connectivity (80). Despite the controversy around direct clinico-radiological correlations (81), some studies in ALS have reported significant associations, most often with functional measures (73, 82–84), disease duration (59, 73), and progression rates (20, 40, 85).

### Practical Limitations

For a condition in which accumulation of physical disability is accompanied by ventilatory compromise with orthopnoea, supine MRI limits longitudinal assessment to those with slower rates of progression (39). The application of such a biomarker as an outcome measure in a small-scale clinical trial would then entail costly statistical compromises, since no ideal solution exists for the imputation of data points selectively lost from those patients with more aggressive disease (86).

## Encephalography

Cortical processes, and the diseases that impact on them, are inadequately described without reference to dynamic neural communication (87, 88), but this necessitates temporal precision, without the dispersive effects of the haemodynamic response function that smears neural signals across several seconds (89). Surface electroencephalography (EEG) as a biomarker in ALS is appealingly practical, well tolerated and non-invasive.

### Methodological Limitations

Even a high-density array of surface EEG electrodes still sacrifices spatial resolution owing to the attenuation and mutation of neural signals as they pass through several tissue layers with varying electrical conductivity (89). Magnetoencephalography (MEG) permits recording of tiny (femtoTesla) fluctuations in the magnetic field external to (and undispersed by) the scalp (90). Yet reconstruction of cortical sources remains a mathematically “ill-posed” problem—any given recorded signal could in theory be generated by multiple neural sources and the analytical choice to address this (for example “beamforming”) necessitates certain assumptions (91). MEG's improvement in spatial precision is also offset by expenses and susceptibility to artifact from ferromagnetic interference, albeit mitigated by acquisition and analysis standardization (92, 93). The resulting data is feature-rich, subsequent analysis may necessarily be restricted to a frequency-band of interest or a selected connectivity metric, these choices may in turn influence study conclusions (94) (Table 2).

**Table 2:** Selected motor task-based and resting-state encephalographic studies in ALS.

**Authors**	**Year**	***n***	**EEG/MEG (channels)**	**Protocol**	**Main measure**	**Phenotype correlations**
Westphal et al. (104)	1998	16 ALS 16 HC	EEG (11c)	Self-paced R fist closure	Reduced BP	Spasticity correlation
Thorns et al. (105)	2010	13 ALS 13 HC	EEG (19c)	Cued R or L index finger button press	Reduced BP	N/A
Inuggi et al. (107)	2011	32 ALS 12 HC	EEG (29c)	Self-paced R thumb extension	Reduced MRCPs (only in UMN+ ALS)	Ipsilateral MRCP correlation with movement speed
Riva et al. (119)	2012	16 ALS 15 HC	EEG (29c)	Self-paced R thumb extension	Reduced beta ERS; Unaltered beta ERD	ERS correlation with CST damage via MRI and TMS
Gu et al. (108)	2013	4 ALS 7 HC	EEG (15c)	Imaginary R wrist extension	Slower MRCP rebound	N/A
Bizovičar et al. (120)	2014	21 ALS 19 HC	EEG (30c)	Self-paced R index finger flexion	Reduced beta ERD; Lateralised ERS	None
Proudfoot et al. (122)	2017	11 ALS 9 PLS 12 Presymp 10 HC	MEG (306c)	Cued R or L index finger extension	Excess beta ERD; Delayed ERS	Altered ERS lateralisation in PLS
Proudfoot et al. (126)	2018	17 ALS 11 HC 5 Presymp	MEG (306c)	Cued R and L hand grips	Reduced beta CMC; Reduced inter-hemispheric beta FC	CMC unaltered in Presymp
Mai et al. (143)	1998	18 ALS 14 HC	EEG (18c)	Resting	Reduced central alpha power	Alpha correlation with MRC and Norris scales
Santhosh et al. (144)	2005	12 ALS 12 ALS	EEG (8c)	Resting	Reduced alpha power	N/A
Jayaram et al. (145)	2015	6 ALS 32 HC	EEG (124c)	Resting	Reduced central theta power; Widespread increased high gamma power	Gamma reduced only in patient with ALSFRS = 0
Iyer et al. (147)	2015	18 ALS 17 HC	EEG (128c)	Resting	Increased FC especially within salience and default-mode networks	N/A
Nasseroleslami et al. (148)	2017	100 ALS 34 HC	EEG (128c)	Resting	Increased FC especially interhemispheric theta and fronto-parietal gamma	FC correlation with structural MRI degeneration
Fraschini et al. (150)	2018	21 ALS 16 HC	EEG (61c)	Resting	Widespread reduced alpha FC	N/A
Proudfoot et al. (151)	2018	24 ALS 24 HC 15 Presymp 9 PLS	MEG (306c)	Resting	Widespread increased broadband FC	Similar changes in PLS. More subtle changes in Presymp.
Sorrentino et al. (154)	2018	50 ALS 25 HC	MEG (163c)	Resting	Broadband increased FC with disorganized topology	Advanced ALS associated with a more centralized, “vulnerable” network

*HC healthy control, FC functional connectivity, Presymp asymptomatic ALS-causing gene carriers, BP Bereitschaftspotential, MRCP movement related cortical potential, ERD event related desynchronization, ERS event related synchronization PLS primary lateral sclerosis, CMC cortico-muscular coherence UMN+ above average quantity of upper motor neuron signs, CST cortico-spinal tract, ALSFRS ALS functional rating scale (disability metric)*.

### Evoked Potentials

Small-scale EEG studies have addressed the utility of somatosensory, visual and brainstem evoked potentials in ALS (95–100). Reflecting the inconsistency of reported results, these well-established and standardized assessments have failed to find any routine clinical application in ALS, although they may yet find a role in multimodal assessment (101, 102).

To better reflect the pathological burden in ALS studies have therefore moved toward either motor or cognitive activation paradigms, initially appraising cortical processes via evoked response potentials [ERPs, previously reviewed in (103)]. The “Bereitschaftspotential,” a classical lateralized change in cortical electrical potential, easily recordable during movement preparation, appeared robustly decreased in ALS (104, 105). More recent studies have considered the implications of abnormal movement-related cortical potentials (MRCPs) in ALS in terms of clinical and structural correlates. While a study of 21 ALS patients demonstrated higher MRCPs overall, the effect was shown to be driven by patients with a low burden of clinically detectable UMN morbidity (106). The inference that increased MRCPs reflect cortical compensatory mechanisms was born out by longitudinal study of a sub-set in whom MRCPs declined over 10 months. A comparable study of finger movement in 32 ALS patients revealed reduced MRCPs only in patients with a high UMN burden, alongside evidence of ipsilateral premotor activation to suggest a compensatory “boundary shift” (107). MRCPs are also elicited during imagined movements, but only a limited study in ALS has thus far been performed (108), mandating replication before application of these measures in control and communication devices is to be seriously considered.

### Motor Paradigms

Motor events (including self-generated movement) are reflected in frequency-specific changes to continuous “background” neuronal oscillations (109). As might be expected, the neurodegeneration associated with ALS results in distinct alteration to pre-central sensorimotor rhythms. While studies are yet to be widely replicated, they show promise both in terms of relevance to daily motor tasks, and sensitivity to detect early cortical dysfunction in patients still capable of performing the task in question. The results may also contribute to the ongoing efforts to characterize a presymptomatic phase to ALS and have implications for the development of brain-computer interfaces aiming to facilitate environmental control by patients with advanced ALS (110).

Movement is accompanied by reliable and well-characterized fluctuations in neural signal power, particularly within the beta (15–30 Hz) band, with recognizable anatomical localization to motor cortex. Beta-band power is reduced (event related desynchronization, ERD) prior to and during movement execution; movement termination is followed by an equally reliable increase in power well above baseline levels (synchronization, ERS or post-movement beta-rebound) (111). Temporally corresponding to fluctuations in cortical excitability (112), ERD and ERS are adjusted to meet task requirements [including force (113), speed (114), and complexity (115)], are sensitive to pharmacological manipulation [particularly synaptic GABA levels via benzodiazepines (116) or tiagabine (117)] and may be disrupted by other disease states including Parkinson's (118).

### Motor Studies (EEG)

Two independent EEG studies have demonstrated attenuation of ERS in ALS. The first involved 16 patients efficiently performing self-paced thumb extensions (119). The degree of ERS attenuation was shown to correlate with corticospinal pathological burden as measured by both mean diffusivity on structural MRI and diminished motor evoked potentials in APB in response to TMS stimulation. The second study included 21 patients performing both sniffing and right index finger flexion (120). Although the patients had detectable weakness in terms of both maximal grip strength and sniff nasal-inspiratory pressure, there were no group differences in the precise pressure produced during the task performance. Neural data from the sniff task were heavily contaminated by facial muscle artifact, but the finger flexion task resulted in reliable ERD/S. The ALS patients were observed to have diminished beta ERD, interpreted as a consequence of pyramidal cell degeneration. Both motor preparation and execution timepoints were affected, while the lateralization of beta ERS was also altered. The study failed to establish clinical correlations with these measures, nor was there any successful correlation with F-wave elicitability (an imperfect measure of corticospinal tract integrity in any case).

### Motor Studies (MEG)

The neural signal acquired by MEG is far less susceptible to distortion as it passes through skull and scalp, source modeling is therefore likely to be more accurate than EEG, and an expanding range of MEG studies have specifically appraised sensorimotor rhythms (121). A MEG study involving 11 ALS patients, 9 with PLS, and 12 asymptomatic genetic mutation carriers, investigated sensorimotor rhythms during a laterally-cued motor preparation task requiring speeded index finger extension of either hand (122). Whole-brain source-space data were analyzed pre, during, and post movement, specifically focusing on beta-band frequencies. Although the task was behaviorally performed comparably by ALS patients, the neural data revealed larger beta ERD, 500 ms after cue presentation, during the period of maximal motor preparation, particularly within contra and ipsilateral gyri. Beta ERS, after movement termination, was delayed in both patient groups. The asymptomatic carriers produced excessive beta ERD during motor execution. Conceptually the results are concordant with cross-modality support for cortical hyperexcitability in ALS (123, 124).

The integrity of upper motor neurone pathways can also be non-invasively appraised using MEG. Cortico-muscular coherence (CMC), by which neural oscillations and surface electromyography correlate temporally (particularly during sustained contraction), principally reflects direct corticospinal drive to the peripheral musculature (125). A MEG study of 17 ALS patients was designed to measure CMC during a bilateral forearm grip task (126). As expected, source-space beta CMC was distinctly strongest from the contra-lateral precentral gyrus, but this frequency specific peak was markedly attenuated in the ALS group, despite adequate grip production and without any correlation to force production. The analysis also took advantage of MEG spatial precision to consider motoric cortico-cortical communication during the same task performance. Interhemispheric functional connectivity, in terms of beta band amplitude envelope correlation, was reduced in ALS patients. The inference of reduced CMC, a measure that in health indexes the quality of motor performance (127), is that beta coherence may serve as a novel UMN specific biomarker at the disposal of future therapeutic efforts (128).

### Extra-Motor Studies

Taking advantage of the high temporal resolution of encephalographic data, component steps in the complex cognitive dysfunction associated with the ALS-FTD syndrome may be examined. The mismatch negativity (MMN) paradigm considers the attentional modulation of auditory perception. An early EEG study failed to show any abnormalities within ALS patients (129). However, using MEG, plus subtle experimental design adjustments in 12 participants all with bulbar symptoms, MMN response amplitudes were shown to increase relative to healthy controls (130). Given the previously demonstrated sensitivity of MMN responses to ketamine administration, the authors tentatively linked their findings to the glutamatergic excitotoxicity ALS pathogenesis theory. This rare example of “gain of function” was not consistently replicated in two later EEG studies, which interpreted delayed MMN responses as evidence of sub-clinical extra-motor dysfunction (131, 132).

Less well-replicated methodologies have also been applied to ALS patients to consider neural processes underlying working memory (133, 134), selective attention (135, 136), and executive control (137, 138). Broadly, these studies have provided further evidence in favor of sub-clinical disruption to “frontal” cognitive processes in keeping with the extended non-motor ALS phenotype (139). Parietal cortex dysfunction was also implicated in an EEG study involving the Wisconsin Card Sorting Test. While 26 ALS patients did not differ in performance of a “set-shifting” task, even patients without mild cognitive impairment failed to produce the expected enhancement of parietal ERPs during a task-switch (140). Although the attenuation of the “switch potential” failed to correlate with neuropsychological indices, the authors speculated whether such sub-clinical deficits could predict future behavioral disorder.

A study requiring cognitive task performance during data acquisition took a very different analytical approach, using 200 s of data to measure “transfer entropy” between scalp electrodes rather than the millisecond granularity of evoked potentials. The directionality of functional connectivity was appraised via EEG in 18 ALS patients, revealing only feedforward (parietal to frontal) connectivity to increase across a broad frequency band (141). As the patients engaged in a spelling task with a view to brain-computer interfacing, sensory (visual) stimuli were hypothesized to be more readily processed in compensation for the diminished proprioceptive input resulting from physical disability, but an alternative explanation in terms of failing cortical inhibition was also acknowledged (20).

### Resting-State Studies (EEG)

The earliest EEG investigations of ALS reflected the emerging concept of cognitive dysfunction within the ALS clinical spectrum, with slowed cortical rhythms noted in non-demented patients (142). A more systematic study of 18 ALS patients conversely revealed sparse differences to healthy controls (143). Only at central electrodes, and only within the alpha band (8–13 Hz), was the power of neural oscillations reduced in ALS. The reduction was interpreted to reflect selective neuronal loss within the sensorimotor cortices. A comparable result was described in a subsequent smaller study (144), and increases in the gamma band (30–90 Hz) power beyond central regions was also reported (145).

Further ALS electrophysiology studies have reflected a growing interest in the so-called “dynome” (146), the extent to which the organization of cortical function is reflected in particular patterns of active connectivity. High-density (128 channel) surface EEG was used to calculate connectivity between both scalp points and projected source nodes in an initial study of 18 patients (147). Fronto-central areas were shown to have increased connectivity, and this was explored across a broad range of measures. A subsequent study expanded this work to 100 patients, including some longitudinal analyses (148), and confirmed EEG-derived connectivity changes in ALS to be more striking than limited group differences in the scalp-recorded power spectrum. This more parsimonious analysis appraised only sensor-space, deriving coherence estimates within 8 consecutive frequency bands. Widespread increases in connectivity were again demonstrated relative to healthy controls, particularly theta band interhemispheric sensorimotor connectivity and gamma band fronto-parietal connectivity. As 59 of the ALS patients had undergone contemporaneous structural MRI, mathematically derived structural “degeneration modes” (accounting for the large-scale gray and white matter changes typical in ALS) were shown to correlate with EEG change, conceptually aligned with the concept of progressive network decline overlying structural disintegration.

Network structures can also be summarized using graph theory metrics, this was explored in sensor-space in 21 patients, demonstrating a more “de-centralised” organization (149). The connectivity metric chosen in this study was phase-based, thus insensitive to any group differences in spectral power, and furthermore was significantly correlated with disability between individuals. This group later re-analyzed the same data reconstructed into source-space (150) and filtered into 3 classical frequency bands to show spatially distributed decreases in connectivity, albeit restricted to the alpha band spectrum.

### Resting-State Studies (MEG)

A resting-state MEG study explored functional connectivity in 24 ALS patients using source-space data acquired after co-registration with structural MRI (151). Ten minutes of continuous data was parcellated into 39 regions of interest and the broad-band (3–40 Hz) signal used to calculate “edge” strength between these 39 “nodes,” In keeping with many FMRI studies, functional connectivity was broadly increased in ALS patients relative to age-matched healthy controls, particularly affecting communication links to the posterior cingulate cortex. This finding was aligned with the hypothesis of loss of cortical inhibitory neuronal influences underlying cortical excitability in ALS (5). Comparable posterior non-motor connectivity changes were described using FMRI (152). Nevertheless, the diversity of reported results and interpretations serves to highlight a need for replication and standardization between centers and where possible across modalities (153). A further study of 50 patients, using a different (phase-based) connectivity measure, also described widespread connectivity increases in ALS (154). The increases were not restricted to specific frequency bands and the extracted graph theory metrics suggest global network hyper-centralization to accompany disease progression.

## Future Directions

MEG is providing broader insight into cognitive mechanisms underpinning higher cortical function in health (155), and comparable results may eventually prove achievable using surface EEG (156). The next generation of wearable sensors may yet dramatically expand MEG's application (157). The spinal cord is a core but functionally understudied aspect of the motor system disintegration that characterizes ALS. Spinal FMRI is in its infancy (158), but a number of promising studies have already been published in animal models (159, 160), healthy populations (161, 162) and other clinical cohorts (163, 164). The goal of non-invasively studying the integrated activity of upper and lower motor neurone pools looks more feasible with the success in studies involving the dorsal pathways (165). Cerebral FMRI parameters are likely to take an increasing role in emerging machine-learning and classification studies both in diagnostic and prognostic applications (67, 76). Future studies need robust longitudinal design and to capitalize on the growing infrastructure for multicentre studies. This will permit the testing of pathogenic hypotheses within larger cohorts of clinically more homogeneous ALS patients, and define the earliest markers of pathology in presymptomatic individuals essential for the assessment of future neurotherapeutic interventions.

## Author Contributions

All authors listed have made a substantial, direct and intellectual contribution to the work, and approved it for publication.

### Conflict of Interest Statement

The authors declare that the research was conducted in the absence of any commercial or financial relationships that could be construed as a potential conflict of interest.
